# Books Reviewed

**Published:** 1868-05

**Authors:** 


### Editorial Department.

### Books Reviewed.

A Practical Treatise upon the Diseases of Women, by T. Galilard Thomas, M. D. Henry C. Lea: Philadelphia.

A great deal is being said and written upon the Diseases of Women; and physicians everywhere appear desirous of shedding new light upon this department of medical practice. We have examined this work by Dr. Thomas upon most of the points in controversy, and take this opportunity to announce that physicians will find everything scientific and useful in this department, described and represented far better than they can do it themselves, if they try, and perhaps until the art has made some more progress, had better not try to add to, or change the text, as given us by this author. We do not mean to say that the subject is closed, but the discussion is as complete as present knowledge will permit. Dr. Thomas' ideas are doubtless as correct as any ones, and his manner of communicating them to others is admirable. Two hundred illustrations add very much to the value of the work, and leave, in this direction, nothing more to be desired.

The Diseases of Women, by T. Gaillard Thomas, M. D., is a book not only to be owned, but to be read and followed. Perhaps this is saying a little too much for a book—books are not to be followed with unreasoning adhearance; they are guides, which point us the right way, but every true physician seeks in some respects his own path; certainly in the diseases of women he must be allowed to take his own way. We regard the work as an embodiment of the present knowledge in this department, and as eminently deserving the confidence of the profession.

---

Atlas of Venereal Diseases. By A. Cullerier, Surgeon to the Hospital Du Dieu, Member of the Surgical Society of Paris, etc. Translated from the French, with notes and additions, by Freeman J. Bumstead, M. D., Professor of Venereal Diseases in the College of Physicians and Surgeons, New York, etc. With one hundred and fifty beautifully colored figures on twenty-six plates. Part 1, 2. To be completed in five parts. Philadelphia: Henry C. Lea; 1868. Price, $3.00 per Part.

We would direct the attention of our readers to Parts 1 and 2 of the "Atlas of Venereal Diseases," designing to present an extended review when the work shall have been completed. Judging from the parts already received, we predict that this work will be the most elaborate and practical treatise ever presented upon venereal diseases. Its author is favorably known as a specialist in this department, while the name of Dr. Bumstead, the translator and editor, enjoys the highest reputation as a careful observer and close student, both at home and abroad.

The introduction of the work is devoted to a discussion of how to study syphilis; its history, evidence, contagion, evolution, inheritance, pathological anatomy and treatment. Parts 1 and 2 discuss the subjects of Blennorrhagia and Blennorrhœa, as these diseases occur in the male and female, together with their various complications. The chromo-lithogrophic illustrations representing the parts as they appear in these diseases are accurate and true to nature, surpassing any similar representations we have yet seen in this department.

---

Signs and Diseases of Pregnancy. By Thomas Hawkes Tanner, M. D., F. L. S. Philadelphia: Henry C. Lea.

This work is comprised in twelve chapters, upon the following subjects, making a volume of about five hundred pages: General observations on the state of Pregnancy; Signs and symptoms of Pregnancy; the Diseases which simulate Pregnancy; the Duration of Pregnancy; the premuture expulsion of the fœtus; the examination of substances expelled from the uterus, etc.; extra-uterine gestation; superfœtation—missed labor; the diseases which may coexist with pregnancy and reciprocal influence; the sympathetic disorders of pregnancy; the diseases of the urinary and generative organs; the displacements of the gravid uterus. Under these several heads the author has discussed with great minuteness and care all the numerous questions within the scope of the work, and it seems to us that nothing of any interest has been omitted; indeed, it is so complete in this respect that in some cases even the most obviously absurd and inconsistent views are shown to be unfounded, as if physicians might yet believe and adopt them, unless again shown to be irrational. This work sustains the well-known reputation of the author, and contributes to his deserved popularity as an earnest thinker and clear and instructive writer. The illustrations are most excellent, and contribute greatly to the value of the work. Whoever desires to be in possession of all that can be said upon the general questions involved will not fail to possess this work, which is full, complete, and carefully prepared, well worthy attention from medical students and physicians.

---

A Manual of the Dissections of the Human Body. By Luther Holden, F. R. C. S., Assistant Surgeon of and Lecturer on Anatomy at St. Bartholomew's Hospital, London, with notes and additions by Erskine Mason, M. D., Demonstrator of Anatomy at the College of Physicians and Surgeons and Surgeon to the Charity Hospital, New York; illustrated with numerous wood engravings. New York: Robert M. DeWitt, publisher, No. 13 Frankfort street.

The author claims but little originality in the preparation of this work, the material being almost entirely extracted from the standard works on anatomy, compiled and arranged to serve the student in the pursuit of his studies in practical anatomy, and also to refresh the memory of the surgeon in the surgical relation of parts. With this object in view the vessels, nerves, lymphatics, muscles, etc., are described as they present themselves in each region upon dissection, their surgical bearings especially pointed out, and wherever practicable and consistent with the character of the work, operations upon the cadaver described. The editor has made such additions relating to the anomalies of vessels and muscles, as in his opinion, the student should become acquainted with, also added the measurements and weights of organs. One hundred and thirty-four illustrations of unusual merit embellish the work, and greatly add to its value. Altogether the work of Dr. Holden is one of the best and most perfect anatomical guides we have yet seen, and its merits cannot be too highly commended; it is neither too concise nor too diffuse. The publisher has furnished the work in a highly creditable manner.

